# Gender Differences in Physical Activity, Sedentary Behavior and BMI in the Liberec Region: the IPAQ Study in 2002–2009

**DOI:** 10.2478/v10078-011-0029-6

**Published:** 2011-07-04

**Authors:** Sigmundová Dagmar, Sigmund Erik, Frömel Karel, Suchomel Aleš

**Affiliations:** 1 Center for Kinanthropology Research, Faculty of Physical Culture, Palacky University, Olomouc, Czech Republic; 2 Department of Physical Education, Faculty of Sci., Humanities, and Education, Technical University in Liberec, Czech Republic

**Keywords:** questionnaire, overweight, obesity, trends, physical activity

## Abstract

The occurrence of overweight, obesity and physical activity (PA) belongs to major factors influencing health. However, information on the longitudinal development of PA in Europe has been, up until now, insufficient. The aim of the study was to describe the changes in PA, sedentary behavior and BMI in the inhabitants of the Liberec region during the surveybetween 2002 – 2009. The data were obtained in 2002–2004 using the short version of the IPAQ questionnaire, in 2005–2009 using the long version of the IPAQ. The sample of participants comprised 957 males and 1066 females aged 25–60 years. A significant decrease in sitting accounting for minutes on working days has been recorded both in men and women. A significant increase of PA both in men and women was reported, however the interpretation of the increase needs to be done cautiously due to the application of the short and long version of the IPAQ. Regarding PA intensity, men show more PA in total than women, which can be explained mainly by the differences in vigorous PA. Women show significantly lower values of BMI in all years in which surveys were conducted than men (p<0.001). Based on self-reported data, we have recorded an increase in the number of overweight and obese people. Time spent sitting on working days has had a tendency to decrease, yet a negative trend in the decrease in self-reported total PA has not been confirmed. Despite the fact that there has been an increase in self-reported PA, no decrease in BMI was registered.

## Introduction

To lower the number of overweight and obese people and to increase physical activity are among the main goals of public health research(Oja et al., 2010; U.S. Department of Health and Human Services, 2000; U.S. Department of Health and Human Services, 2009; WHO, 2004). The effect of the negative development of physical activity and the increase in physical inactivity related to increasing BMI have been an issue all over the world (Avenell et al., 2004; Guthold et al., 2008; Knai et al., 2007; Talbot et al., 2003). Regarding health benefits, two thirds of the inhabitants of the European Union did not reach, in the year 2002, the recommended level of physical activity (Sjöström et al., 2006).

However, it is difficult to determine trends in physical activity across Europe on the basis of irregularly updated data on the levels of PA in individual countries. The lack of data from regularly repeated surveys across the European region prompts the need for regular and repeated monitoring of physical activity on national and regional levels (Cavill et al., 2006).

Trends in physical activity are related to the age of participants, to individual types of physical activity and to meeting health related recommendations (Cavill et al., 2006; Haskel, 2009; Haskell et al., 2007; Hardman & Stensel, 2009; Oja et al., 2010; Saris et al., 2003). A decrease in physical activity in young adults was recorded in the Amsterdam Longitudinal Growth and Health Study (Van Mechelen et al., 2000). A study of Finnish adults, which was carried out in 1972–2002, on the other hand, showed that physical activity tended to increase with age (Borodulin et al., 2007). An Australian cross-sectional study points at a decrease in meeting physical activity recommendations, especially in socioeconomically disadvantaged areas (Merom et al., 2006). A decrease in regular vigorous physical activity; regular, sustained physical activity, and an increase of inactivity were recorded in a study of the U.S. population aged 15–64 (Caspersen et al., 2000).

Physical activity is closely related to weight assessed on the basis of the BMI. Although higher BMI does not necessarily equal a higher proportion of body fat, BMI still is a simple and recognized criterion which assesses overweight and obesity (Bouchard et al., 2007).

Obesity and overweight in the adult population ranges in between 28–79% in European countries.

Since the 80’s, an increase in obesity has been recorded also in adults in countries in which the level was previously below 5%. For example, in Ireland, England and Scotland the incidence of overweight has risen rapidly, by more than 0.8% per year (Branca et al., 2007). An increase in BMI by 2 kg/m^2^ during the 11 years of follow-up was also recorded in the HUNT study in the Norwegian population (Sund et al., 2010). A negative trend has been also recorded in the Czech Republic, where overweight and obesity has been identified in 81% of male and 71% of female population aged 45–69 in 2002–2005 (Pikhart et al., 2007). Kunešová (2006) claims that overweight and obesity reached 52% in 2005 in the Czech adult population and, as opposed to a survey carried out in 2000/2001, an increase of 3% was found. When monitoring health related behavior, we need to consider gender differences. For example, leisure time physical activity is associated with social functioning only in women (Tessier et al., 2007). Men are more physically active than women (Florindo et al., 2009). The differences between physical activity in men and women are caused by different lengths of time spent performing moderate and very vigorous activities (Van Mechelen et al., 2000). Walking is a better predictor of meeting health criteria in men and women than other factors (Cole et al., 2006).

The Liberec region is one of 14 regions in the Czech Republic. It is located in the Northern part of the country, bordering with Germany and Poland. It is a region with a number of historical monuments, cultural and sport facilities. The town of Liberec is considered to be a centre for sports (the White Tigers arena, the ski resort in Harrachov, and the Aquapark in the Babylon Entertainment Center) and for education (The Technical University, Liberec). The Liberec region with its 439,000 inhabitants is a region with a higher proportion of children than the elderly. About 12.5% of adults living there have been diagnosed as obese. The mortality rate decreases longitudinally. Cardiovascular diseases are the most frequent cause of death in the Liberec region, but there is a progressive development of the support of a healthy lifestyle in the region (Czech Statistical Office – Liberec, 2010; Liberecký kraj, 2010; Ústav pro ekopolitiku, 2005; Úzis ČR, 2010).

The Czech Republic, similarly to other post-communist countries, tends to copy the negative development of a decrease in physical activity and the increase in overweight and obesity of economically developed countries (Branca et al., 2007). Countries in Central and Eastern Europe can learn from the negative Western European and global experience (Knai et al., 2007). Due to the different characteristics of each region in the Czech Republic, it is essential to take into account these specifics when examining physical activity behavior in relationship to obesity and overweight. When assessing physical activity, sedentary behavior and BMI, the different social roles of men and women should be also considered.

The aim of this study was to describe the changes in physical activity, sedentary behavior and BMI of the inhabitants in the Liberec region in the years 2002 – 2009, in which surveys were conducted and, further, to describe the gender differences in physical activity, sedentary behavior and BMI in this region.

## Material and Methods

### Ethics

This study was carried out as part of nationwide research on physical activity that is in turn included in international research, i.e.: the International Physical Activity Questionnaire Prevalence Study, the International Physical Activity and the Environment Network (IPEN).This study was approved by the Institutional Research Ethics Committee at Palacky University.

Participation was voluntary; participants received no incentives and could withdraw from the study at their own free will. The study objectives were provided to participants, and each participant signed an informed consent form for inclusion in the study. All data were anonymous and confidential.

### Procedures and Participants

Questionnaires were distributed randomly in the Liberec region, using trained distributors. Data for this sectional study were collected in 2002–2004 using the short IPAQ questionnaire, and in 2005–2009 using the long IPAQ questionnaire. The survey was carried out in Spring and Fall under very similar climactic conditions.

In the survey of 2002–2004, 1649 questionnaires were collected from respondents aged 15–69. 542 were excluded due to being too young or too old or because of incomplete or incorrectly filled out questionnaires. The final analysis for the 2002–2004 period included 1077 adults (521 men and 556 women) aged 25–60. In 2005–2009, 1572 randomly selected adults aged 15–69 participated in the study. Out of this number, 626 questionnaires were excluded from the sample due to either the participants being too young or too old or due to incompletely or incorrectly filled-out questionnaires. The final analysis included 946 adults (436 men and 510 women) aged 25–60. In total, the analysis presented in this paper comprised data from 2023 participants (957 males and 1066 females). Participants’ ages ranged from 25 to 60 years of age (mean 40.18 years, SD=9.56).

### Instruments

The short IPAQ is an internationally standardized short administrative version of the questionnaire for the estimation of the level of physical activity in the last 7 days (Craig et al., 2003, Frömel et al., 2004). The long and the short IPAQ are useful instruments for the estimation of the level and the amount of physical activity, especially in epidemiological studies.

The questionnaires were designed to be used by adults aged 18–65 yrs. The short version provided information on the time spent walking, in vigorous- and moderate intensity activity and in sedentary activity. Participants were instructed to refer to all domains of physical activity. The long version was designed to collect detailed information within the domains of household and yard work activities, occupational activity, self-powered transport, and leisure-time physical activity as well as sedentary activity (Craig et al., 2003).Vigorous physical activities in IPAQ are activities, that require hard physical effort and significantly increase minute ventilation. Moderate activities in IPAQ are activities that take moderate physical effort and make you breathe somewhat harder than normal. The long version of the IPAQ provides more detailed information on physical activity, yet the value of the estimated total physical activity is higher than in the short version of the IPAQ. Both the questionnaires have acceptable measurement properties (Craig et al., 2003; Meriwether et al., 2006; Sigmund et al., 2009).

The long IPAQ questionnaire was a part of the ANEWS questionnaire (Neighborhood Environment Walkability Scale-Abbreviated; www.ipenproject.org/surveyanews.htm). The IPAQ questionnaires estimate physical activity and inactivity and allow the comparison of vigorous and moderate physical activity, walking and sitting in the context of other personal, demographic and environmental data. The adjustment of data was done in compliance with the internationally recognized methodology of the assessment by the ”IPAQ Research Committee“ (www.ipaq.ki.se). The evaluation of physical activity in METs was 6 METs for vigorous physical activity.

### Statistical Analysis

Statistical analysis was carried out using the software Statistica 8.0. The associations between variables and physical activity were quantified by use of the Spearman correlation coefficient. To assess significant differences, the non-parametric Kruskal-Wallis test and its relevant effect size η^2^ coefficient were used. Commonly used evaluations of values of η^2^ are as follows: 0.06>η^2^≥0.01 small effect, 0.14>η^2^≥0.06 middle-sized effect and η^2^≥0.14 large effect (Morse, 1999).

## Results

When we consider physical activity in relation to age groups (age brackets 25–35, 36–45 and 46–60 years), in this sectional study, regardless of the year of monitoring and regardless of gender, there are no significant differences in PA among age groups.

**Figure 1:**
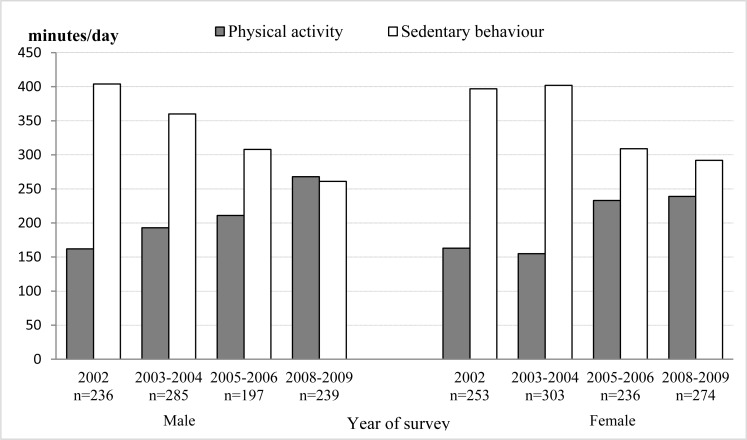
The duration of average weekly physical activity (min/day) and average time spent sitting on working days (min/day).

Over the years when monitoring was carried out (Fig. 1), we have found a decrease in minutes spent sitting on working days both in men [H(3, 957)=75.08; p<0.0001; η^2^=0.079] and women [H(3, 1066)=85.09; p<0.0001; η^2^=0.080]. In the evaluation of total physical activity, we have found a significant increase over the years of monitoring in men [H(3, 957)=54.08; p<0.0001; η^2^=0.057] and in women [H(3, 1066)=54.17; p<0.0001; η^2^=0.051], nevertheless the interpretation of the increase needs to be done cautiously due to the application of the two different types of IPAQ questionnaire. Men show in total,in all the years in which surveys were conducted, significantly more physical activity than women, as assessed in MET-min/week [H (1, 2023)=13.37; p=0.0003; η^2^=0.007].

Based on self-reported data, we have recorded an increase in the number of people with overweight and obesity. In 2002, overweight and obesity was found in 36% of adults aged 25–60, during the survey of 2003–2004 it was already 45%. In the following survey of (2005–2006), we recorded that 43% of the surveyed subjects had overweight or obesity, and in 2008–2009 their proportion increased to 46%.

**Figure 2:**
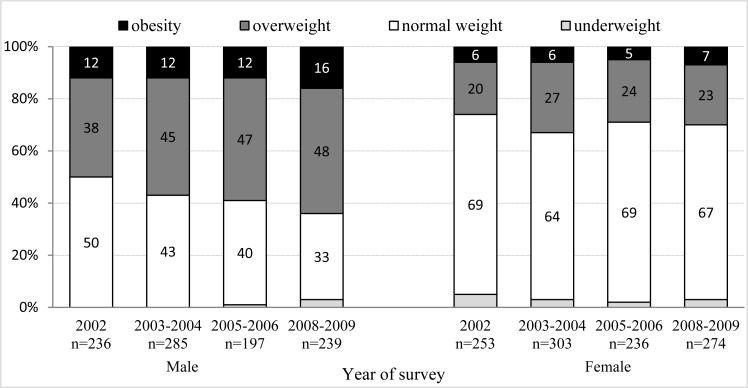
Groups of men and women assessed according to BMI

Women show a significantly lower value of BMI in all the years in which surveys were conducted than men (p<0.001). During individual monitoring, we recorded, in men, a significant increase in BMI [H(3, 957)=13.67; p=0.003]. In women (Fig. 2), the value of BMI is almost equal (p=0.27) in particular years in which surveys were conducted (2002, 2003/2004/2005/2006 and 2008/2009).

**Figure 3:**
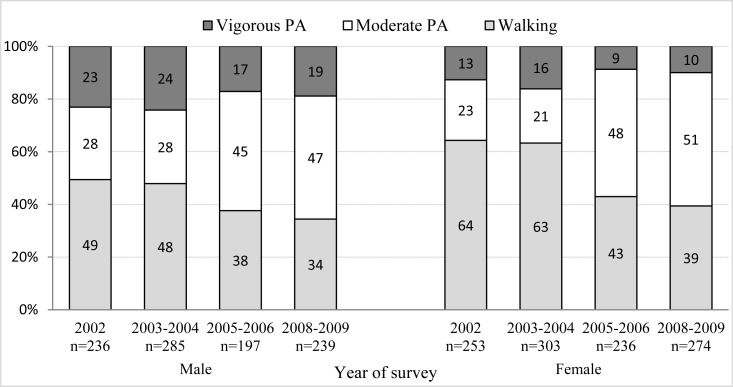
The proportion of physical activity (PA) in percentages according to its vigorous intensity in men and in women in the individual

In all years in which surveys were conducted, significant differences were found in vigorous physical activity between men and women (p<0.05) in favor of men (Fig. 3). In 2002 [H_y2002_(1, 588)=17.91; p=0.0001; η^2^=0,031] and in 2003/2004 men showed a significantly higher degree of moderate physical activity than women [H_y2003/4_(1, 489)=17.70; p=0.001; η^2^=0.036]. On the other hand, in 2005/2006 women showed more moderate physical activity than men [H_y2005/6_(1, 433)=4.57; p=0.03; η^2^=0.011]. Significantly more walking was found in women than men in 2002 (p<0.0001) and 2005/6 (p=0.005). In all the years in which surveys were conducted walking in men and women was at an equal level.

## Discussion

Overweight and obesity are the main risk factors of current civilization diseases. The trends in the development of BMI are relatively well documented (Avenell et al., 2004; Guthold et al., 2008; Knai et al., 2007), however, information about the present development of physical activity in Europe has been scarce until recently (Cavill et al., 2006). The lack of a repeated survey across the European region points to the need of a physical activity survey in this population (Cavill et al., 2006). The main aim of this study was to characterize the changes in physical activity, sedentary behavior and BMI of the inhabitants of the Liberec region and its surroundings 2002 – 2009.

As opposed to expectations, a decrease in the time spent sitting on working days was found in this study both in men and women. The Center for Disease Control and Prevention (2005) analyzed data for the period 1994–2004 and found that the prevalence of leisure-time physical inactivity declined and, in the year 2005, almost one quarter of the U.S. adults reported no leisure-time physical activity. Similarly, results of the Baltimore Longitudinal Study of Aging show a decrease in the number of sedentary male adults (Talbot et al., 2003). In a Polish study, they found that approximately 35% of Polish adults are not physically active in their leisure time (Drygas et al., 2009). However, the prevalence of a sedentary lifestyle (defined on the basis of low energy expenditure) in Europe is high and in adults aged 25–64 it is, on the average, apparent in 59–64% of that population (Varo et al., 2003).

Continually, with the decline in sedentary behavior, we recorded an increase in total physical activity. This increase could have been, however, due to the transition to the application of the long IPAQ version instead of the short one. With the long one, due to more detailed questions; we obtained higher values of total physical activity than with the short version (Ainsworth et al. 2006; Sigmund et al., 2009). This increase in vigorous physical activity was found in the U.S. population only in men (Talbot et al., 2003). In a Finnish study, they also found that physical activity tended to increase with age (Borodulin et al., 2007). Due to biological mechanisms, a decrease of physical activity with age is an expected trend (Sallis, 2000). This fact was also confirmed in adult people in the U.K. (Miles, 2007). If we consider the intensity of the performed physical activity in this study, men are more physically active than women. Similar results were also found in Brazil (Florindo et al., 2009), in member states of the Gulf Cooperation Council (Mabry et al., 2010) in Jordan (Ammouri et al., 2007) in Turkey (Karaca et al., 2009) in China (Yan et al., 2007), in the Czech Republic (Suchomel et al., 2008) or in Poland (Drygas et al., 2009).

Nevertheless, similarly to the Amsterdam longitudinal study, the differences in physical activity between men and women are caused by different amounts of time spent in moderate and vigorous activities (Karaca et al., 2009; Van Mechelen et al., 2000).

Despite a decrease in the amount of time spent sitting and an increase in physical activity both in men and women, it is not reflected in a decrease in BMI. We can see the positive influence in women when there is no significant increase of BMI. In total, we recorded an increasing number of adults with overweight and obesity in the population in this study. Similar trends are found in those European countries where there has been an increase of obesity since the 80s (Branca et al., 2007). An increasing trend in obesity and overweight was also confirmed in Eastern Europe (Knai et al., 2007). This study, carried out in 2008–2009, found overweight and obesity in almost half of the inhabitants (46%) aged 25–60. While the proportion of people with overweight and obesity found in the Czech Republic in 2005 was 52% (Kunešová, 2006), in the older population (45–69 years old) it was more than 70% (Pikhart et al., 2007). In comparison to European countries, we are somewhere in the middle of this range, with overweight and obesity in the adult population being between 28% and 79% (Branca et al., 2007).

This study has its limitations. Physical activity was assessed using the questionnaire without simultaneous monitoring using an objective technique. The total physical activity in the survey of 2005–2009 could be overestimated due to the application of the long IPAQ questionnaire, which provides more detailed information, especially about vigorous physical activity (Sigmund et al., 2009). When doing intragroup comparison (e.g. men and women) or proportion comparison, this effect is quite eliminated. Due to the fact that these are self-reported data and that the majority of people who agree to participate in this research tend to be more physically active, the results might reflect higher physical activity levels than there truly are. For further research, we recommend the application of monitoring devices as well, e.g. the Actigraph, along with the IPAQ questionnaires, which allows for more accurate estimates of physical activity.

To summarize the results, time spent sitting on working days shows a tendency to decline. In physical activity, we have found an increasing trend, however this could have been caused by the transition from the short to the long IPAQ application. When we consider the intensity of physical activity (MET-min/week), men show significantly higher physical activity. The biggest gender differences in physical activity are found in vigorous physical activity. Time spent walking in women is almost equal to that of men. Women show significantly lower values of BMI than men.The BMI of women in the individual years in which the surveys were conducted is equal, whereas men show increasing BMI over the course of time. Due to the increase in physical activity, when there is not yet a decline in BMI, we conclude that, in order to maintain our appropriate weight, we need to adjust our entire lifestyle. Due to the lack of data about physical activity in Europe, it is necessary to carry out studies with repeated monitoring of physical activity in populations in Europe.
